# Extracellular Vesicle Isolation and Characterization from Periprosthetic Joint Synovial Fluid in Revision Total Joint Arthroplasty

**DOI:** 10.3390/jcm9020516

**Published:** 2020-02-14

**Authors:** Julian M. Rüwald, Thomas M. Randau, Cäcilia Hilgers, Werner Masson, Stephan Irsen, Robin L. Eymael, Hendrik Kohlhof, Sascha Gravius, Christof Burger, Dieter C. Wirtz, Frank A. Schildberg

**Affiliations:** 1 Clinic for Orthopedics and Trauma Surgery, University Hospital Bonn, 53127 Bonn, Germany; 2 Electron Microscopy and Analytics, Center of Advanced European Studies and Research, 53175 Bonn, Germany; 3 Medical Faculty, University Hospital Essen, 45147 Essen, Germany; 4 Department of Orthopaedics and Trauma Surgery, University Medical Center Mannheim of University Heidelberg, 68167 Mannheim, Germany

**Keywords:** EVs, extracellular vesicle isolation, periprosthetic joint synovial fluid, periprosthetic joint infection, aseptic implant failure

## Abstract

Extracellular vesicles (EVs) comprise an as yet insufficiently investigated intercellular communication pathway in the field of revision total joint arthroplasty (RTJA). This study examined whether periprosthetic joint synovial fluid contains EVs, developed a protocol for their isolation and characterized them with respect to quantity, size, surface markers as well as documented their differences between aseptic implant failure (AIF) and periprosthetic joint infection (PJI). EV isolation was accomplished using ultracentrifugation, electron microscopy (EM) and nanoparticle tracking analysis evaluated EV presence as well as particle size and quantity. EV surface markers were studied by a bead-based multiplex analysis. Using our protocol, EM confirmed the presence of EVs in periprosthetic joint synovial fluid. Higher EV particle concentrations and decreased particle sizes were apparent for PJI. Multiplex analysis confirmed EV-typical surface epitopes and revealed upregulated CD44 and HLA-DR/DP/DQ for AIF, as well as increased CD40 and CD105. Our protocol achieved isolation of EVs from periprosthetic joint synovial fluid, confirmed by EM and multiplex analysis. Characterization was documented with respect to size, concentration and epitope surface signature. Our results indicate various differences between PJI and AIF EVs. This pilot study enables new research approaches and rising diagnostic opportunities in the field of RTJA.

## 1. Introduction

The periprosthetic joint infection (PJI) is a frequent and devastating complication following knee or hip arthroplasty with dramatic impact on patients and healthcare systems [1,2,3]. The prospectively important distinction between PJI and aseptic implant failure (AIF) remains challenging and often requires an ensemble of various diagnostic tests due to the lack of a gold standard [3,4,5]. Consequently, much effort has been made to explore new PJI biomarkers over the last years [6,7]. Many of these target the intercellular communication including C-reactive protein (CRP), soluble intercellular adhesion molecule-1 (sICAM-1), procalcitonin, interleukin-6 (IL-6), tumor necrosis factor α (TNFα) and other inflammatory cytokines within the serum or joint aspirates [8].

In recent years, extracellular vesicles (EVs) have emerged as a novel cellular communication pathway. Since the first description of exosomes in the 1980s as a vehicle to dispose cellular waste, scientific convergence has evolved to attribute enormous diagnostic potential to nanoscale vesicles as multimodal reflectors of a cell’s (patho-)physiological state [9,10]. These properties have dubbed EVs “liquid biopsies”, which are released into the extracellular space by multicellular organisms with enclosed proteins, lipids and nucleic acids as their cargo to act on distant receiver cells in form of exosomes (30–100 nm) and microvesicles (50–1000 nm) or, in case of cell death, apoptotic bodies (1000–5000 nm) [11,12]. Lipid-bilayer nanovesicles have been isolated from various biological fluids including synovial fluid of patients with rheumatoid- and osteoarthritis [13,14,15,16]. Intriguingly, immune regulatory functions are an established property of EVs and it has been shown that bacterial infections can elicit the immunologic release of nanovesicles with distinct molecular characteristics [17,18,19].

Within the field of revision total joint arthroplasty (RTJA) EVs comprise an as yet uninvestigated entity. Therefore, this study aims to isolate and characterize EVs from periprosthetic joint aspirates with respect to their quantity, size, surface epitopes as well as possible variations between PJI and AIF.

## 2. Experimental Section

### 2.1. Patient Collective and Classification

The study was approved by the ethics committee of the University of Bonn and was conducted in accordance with the approved guidelines as well as the declaration of Helsinki. Patients were divided into two groups: PJI was classified according to the 2018 definition of periprosthetic hip and knee infection by Parvizi et al., which delineates major and minor criteria for PJI [4]. Fulfillment of one major criteria, such as the presents of a sinus tract or two positive microbiological cultures of the same organism, was defined as PJI. If major criteria were not met, minor criteria according to a scoring system depicted in Table 1 were evaluated. A score higher than 6 was defined as PJI and confirmed by intraoperatively collected samples. AIF patients did not fulfill these criteria and intraoperatively collected samples showed no evidence of an infection. Routine examinations further included preoperative blood leukocyte counts, serum CRP and joint aspirate cell counts.

### 2.2. Extracellular Vesicle Isolation

Preoperative periprosthetic joint aspirates from either knee or hip were coarsely cleared from cellular components using centrifugation at 240 g for 10 min (Centrifuge 5810 R, Eppendorf) and stored at −80 °C. EV isolation was conducted by differential centrifugation. After thawing, 500 µl of each sample was suspended in 1.5 mL phosphate-buffered saline (PBS). Debris and larger particles were stepwise removed by serial centrifugation (Centrifuge 5430 R, Eppendorf) at 300× g for 10 min, 2000× g for 30 min, and 10,000× g for 45 min. The supernatant was filtered through a 0.45 µm membrane and underwent ultracentrifugation (Optima MAX XP, Beckman Coulter) at 100,000× g for 2 h in an initial step. Afterwards, pellets were resuspended in supernatant-equivalent amounts of PBS and ultracentrifugation was repeated. Pellets were resuspended in 500 µl of PBS and stored at −80 °C for further analysis.

### 2.3. Electronmicroscopy (EM)

Samples for electron microscopy were 5 times diluted in PBS buffer and deposited on holey carbon grids, covered with a 2 nm carbon film (Quantifoil Microtools GmbH, R2/1 + 2 nm C). The carbon grids were rendered hydrophilic by exposing them to an Argon plasma for 2 min (Baltec MED010). Samples were 3 times washed with double-distilled water and then negative stained with 2% aqueous Uranium-acetate solution. Excess solutions were blotted away using filter paper pieces. In the final step samples were dried after blotting using a gentle airflow. Grids were dried for 30 min prior to examination in the transmission electron microscope (TEM). Micrographs were recorded on a CMOS Detector (F416, TVIPS GmbH) using a JEOL JEM-2200F transmission electron microscope (JEOL GmbH). Image were recorded and post-processed using a bandpass filtered with the software tools SerialEM and Imod [20].

### 2.4. Nanoparticle Tracking Analysis (NTA)

Particle size and concentration were estimated by NTA using the NanoSight NS500 (Malvern Panalytical, Software NTA 3.2) with a 532 nm laser and a sCMOS camera. Samples were diluted 1 to 4 with PBS to an appropriate concentration. Four 30 s recordings were captured for each sample. Analysis of the recordings was conducted with detect threshold set to 7, screen gain to 10, blur size and maximal jump distance to automatic. In a secondary analysis, measurement was restricted to the size spectrum of small EVs with a cut-off value of 200 nm. PBS was taken as a control and subtracted from all samples. From four recordings means for the particle size and concentration were calculated for each sample. NTA results were reported for the whole measurable size spectrum as well as the EV subgroup of less than 200 nm.

### 2.5. EV Surface Signature Detection

A commercially available multiplex bead-based EV analysis kit (MACSPlex Exosome Kit, Miltenyi Biotec, Bergisch Gladbach, Germany) was used for the detection of 37 different EV surface markers and 2 isotype controls. This kit consists of 39 types of fluorescently labeled capture beads each coated with antibodies capable of binding to a specific surface epitope. EVs bound by a capture bead were fluorescently labeled with an EV-specific detection reagent. These bead-EV-reagent-sandwich complexes can be cytometrically detected and discerned by fluorescence. For the experiment, the protein concentration mass was determined by a BCA protein assay (Pierce™ BCA Protein Assay Kit, Thermo Fischer, Rockford, IL, USA) as a surrogate of the approximate EV content and adjusted with PBS to equal levels. The multiplex assay was prepared according manufacturer’s “short” protocol using the kit’s filter plate. Samples were analyzed with a MACSQuant Analyzer 10 Flow Cytometer (Miltenyi Biotec) in Express Mode (MACSQuantify^TM^ Software, Miltenyi Biotec) and primary results were generated via automated gating. The blank control was subsequently subtracted. Quantum^TM^ APC MESF beads (Bangs Laboratories, Fishers, IN, USA) were analyzed in the same fashion according to the manufacturer’s instructions. These standardized beads were used to generate a calibration plot for fluorescence quantification (QuickCal. v 2.3, Bang Laboratories).

### 2.6. Statistics

Assuming homogeneity of variances, Mann-Whitney-U testing was used for statistical testing with non-parametric data distribution and calculated with SPSS (SPSS Statistics Version 23, IBM, Ehningen, Germany) with a significance level of 0.05 (* < 0.05, ** < 0.01, *** < 0.001). Graphs were generated using Prism 7 (GraphPad Software, La Jolla, CA, USA). The results are shown either as median with error bars, indicating the interquartile range (IQR), or as mean with the error bars, indicating the standard error of the mean (SEM), as specified in the according figure legend.

## 3. Results

To pursue the question whether periprosthetic joint aspirates contain EVs and if so, to examine whether these differ among AIF and PJI in amount, size or molecular surface, we performed a stepwise multimodal investigation. First, the patient collective was defined and classified by review of the corresponding clinical data. In total, 23 patients (56.5% female) undergoing RTJA at our endoprosthesis center were included. 12 cases fulfilled the 2018 definition for PJI (75% major, 25% minor), 11 cases underwent aseptic revision surgery with negative postoperative analysis of intraoperatively taken samples. The median age was 71.4 years, 73.9% underwent total knee arthroplasty (TKA). The PJI cohort consisted of more males (*p* = 0.036) and had on average a higher Charlsons Comorbiditiy Index (*p* = 0.01, Table 2).

**Figure 1:**
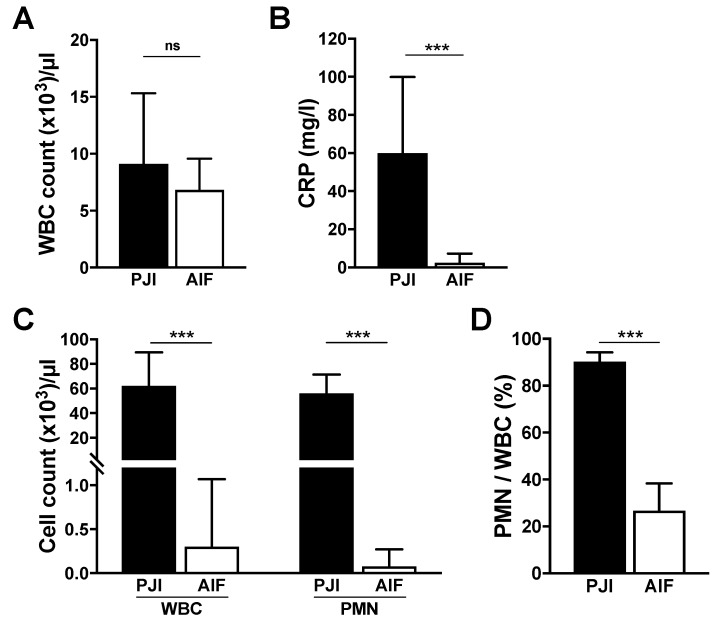
Selected clinical laboratory data of blood and joint aspirates by diagnosis presented as median with error bars indicating the interquartile range (IQR). (**A**) White blood cell count, (**B**) serologic C-reactive protein (CRP) levels, (**C**) white blood cell (WBC) count and polymorphonucleocyte (PMN) count of periprosthetic joint aspirates, (**D**) PMN to WBC ratio in percentage. Mann–Whitney U test with a level of statistical significance of 0.05, * < 0.05, ** < 0.01, *** < 0.001.

Preoperative routine laboratory blood analysis did not show differences in leukocyte counts, while serum CRP was increased for PJI patients (*p* < 0.001). Periprosthetic joint aspirates showed expectably higher overall leukocyte counts (*p* < 0.001) and polymorphonucleocyte to leukocyte ratios (*p* < 0.001) for PJI patients as shown in Figure 1.

**Figure 2:**
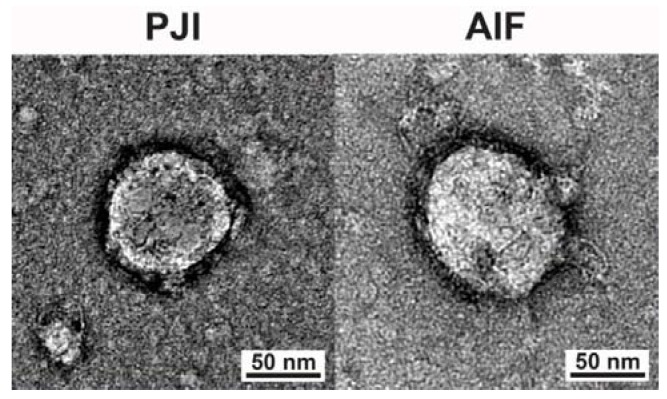
Selected representative electron microscopy images evidencing extracellular vesicle presence in both groups.

To investigate if periprosthetic joint aspirates contain EVs, collected joint fluid samples were enriched for nanoparticles. Electron microscopy was used to facilitate the visual examination of the EV content. Nanoparticle enrichment was achieved using the described differential centrifugation protocol. This method is based on the difference in weight of the components and the stepwise increase of centrifugation speed in order to gradually remove larger cellular debris and particles by collecting and transferring supernatants while discarding pellets. In a final step, samples are subjected to ultracentrifugation, which forces even exceedingly small particles into a pellet that can be recovered and processed further. Electron microscopy revealed vesicular particles and the analysis of size and morphology confirmed, for the first time, the presence of EVs in periprosthetic joint synovial fluid (Figure 2).

**Figure 3:**
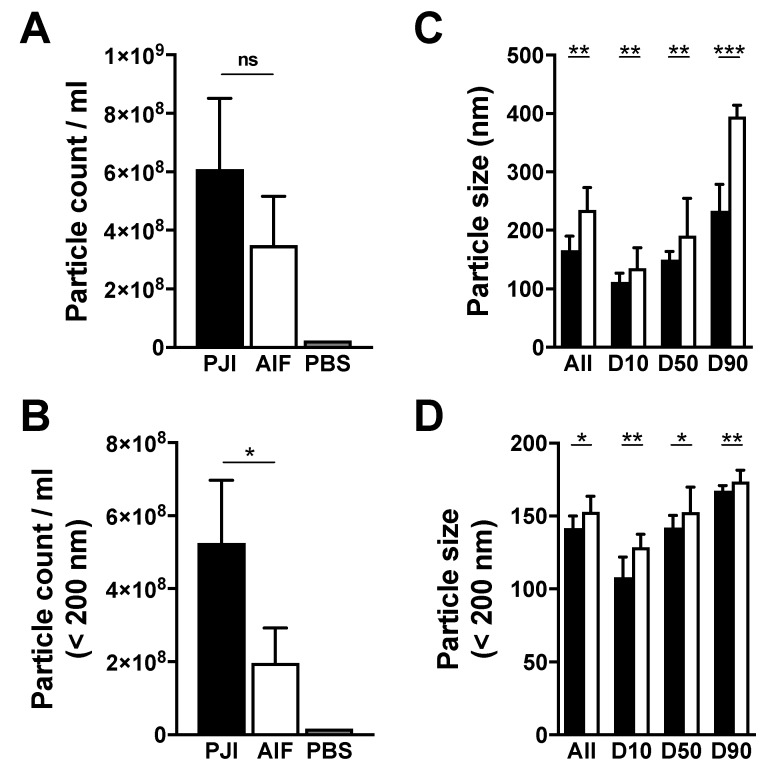
Nanoparticle tracking analysis. Results of the nanoparticle tracking analysis (NTA), presented as median with error bars indicating the interquartile range (IQR) for PJI and AIF, respectively, (**A**) particle count per milliliter (mL) with phosphate buffered saline (PBS) control, (**B**) particle count per mL for particles sized less than 200 nm (small extracellular vesicle (EV) range) with PBS control, (**C**) particle size in nanometer (nm) shown for the complete range and 10th, 50th and 90th percentile, (**D**) particle size in nm within the small EV range (<200 nm) shown for the complete range and the 10th, 50th and 90th percentile; Mann–Whitney U test with a level of statistical significance of 0.05, * < 0.05, ** < 0.01, *** < 0.001.

After obtaining evidence of nanovesicles, we set out to determine their quantity and dimensions. For this, nanoparticle tracking analysis (NTA) was employed, which was able to identify the overall nanoparticle concentrations and sizes within our samples (Figure 3). Additionally, a comparison between both groups showed largely differing amounts and size patterns. These analyses could nicely demonstrate that nanovesicles in aseptic fluid from periprosthetic joint are on average larger with 224.8 ± 90.7 nm compared to samples from infected prostheses with a mean of 156.5 ± 64.4 nm (*p* = 0.001) and tend to be less concentrated (*p* = 0.079). To put focus on the small EV size spectrum, which includes exosomes and smaller microvesicles, but discards a great part of other EVs such as apoptotic bodies, a size cut off at 200 nm was chosen in a second analysis. This showed higher particle concentrations (*p* = 0.032) for PJI samples, while AIF samples displayed greater median particle sizes (*p* = 0.011). Evaluation of the size distribution at D10, D50 and D90—that represent particle sizes at which either 10%, 50% or 90% of the measured particles sizes are below the specified value—confirmed increased particles sizes in AIF samples throughout the whole spectrum (*p* = 0.004, *p* = 0.003, *p* < 0.001).

**Figure 4:**
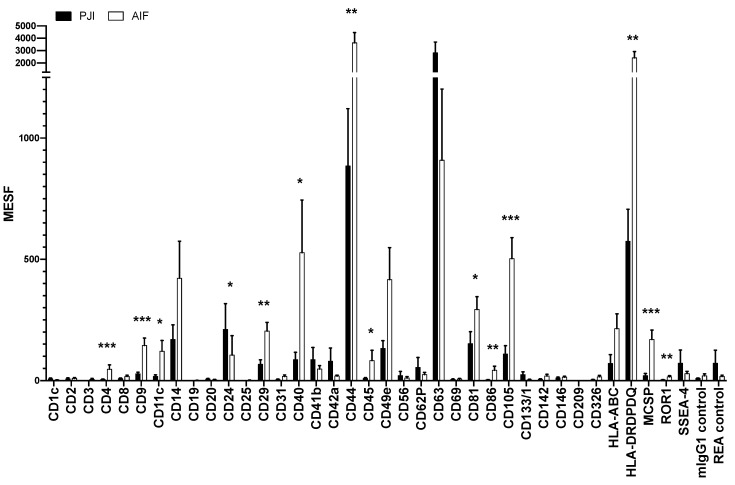
Flow cytometrical analysis of 37 common EV surface epitopes and 2 controls for the PJI and AIF group respectively, presented in molecules of equivalent soluble fluorochrome (MESF) units as mean with error bars, indicating the standard error of the mean (SEM). Statistical analysis with Mann–Whitney U and a statistical significance level of 0.05; * < 0.05, ** < 0.01, *** < 0.001.

Finally, a characterization of the molecular surface signature by analyzing 37 reputable epitopes was executed with a bead-based multiplex flow cytometric assay for EVs to pinpoint possible qualitative differences. Cytometrical analysis of fluorescent antibody-labeled bead complexes generated detectable EV surface signatures. PJI and AIF patients presented distinguishable fluorescence intensity patterns for many of the investigated markers. The results in Figure 4 portray the cytometrical detection of EV-typical surface epitopes CD9, CD63 and CD81 at a minimum of two different surface sides: These capture beads bound particles, which harbored at least one other binding side for the fluorescently labeled antibody reagent, that was added afterwards to the mixture, precluding the possibility of individual antigen capture. High fluorescence intensities for CD63 were measured in both groups, while CD9 and CD81 tended to be more expressed in AIF patients (*p* < 0.001, *p* = 0.037). Besides CD63, the strongest signals were noted for CD44 and HLA DR/DP/DQ, both of which tended to be increased in AIF patients (*p* = 0.004, *p* = 0.002). Likewise, AIF samples surpassed PJI at moderate intensity levels for CD40 (*p* = 0.042) and CD105 (*p* < 0.001). Comparatively higher fluorescence for PJI samples was apparent for CD24 (*p* = 0.023). At low intensities AIF samples tended to express more CD4 (*p* < 0.001), CD11c (*p* = 0.018), CD29 (*p* = 0.008), CD45 (*p* = 0.011), CD86 (*p* = 0.003), MCSP (*p* = 0.001) and ROR1 (*p* = 0.002).

In summary, our results prove the presents of EVs in periprosthetic joint fluid and indicate that these may differ between PJI and AIF in various parameters including their size, amount and surface molecular structure.

## 4. Discussion

Following the first reports of exosomes in the 1980s, further scientific progress has for years been compromised by the lack of awareness for their clinical application potential. The demonstration of their ability to communicate between cells more than a decade later generated broader interest in this field and channeled into the recent surge of EV publications [10]. This development has advanced our basic understanding as much as it has raised new issues. For instance, most experimental designs cannot sufficiently discriminate exosomes from microvesicles in terms of their size, cargo, properties and origin [21]. As a consequence there is broad consensus that most research presents the characteristics of a heterogenous group of nanovesicles (rather than a particular subtype), which may derive from endosomal multivesicular bodies as much as from plasma membrane budding [22]. The formerly exosome-specific tetraspanins CD9, CD63 and CD81 have been proven to be equally present on microvesicles, rendering distinction by these surface epitopes not feasible [9,21]. Therefore, the inflated use of the “exosome” term has largely been replaced by “extracellular vesicles”, acknowledging their diversity.

Another matter under debate is the variety of technologies for EV isolation and analysis, which have classically been strenuous and hampering EV biomarker research in larger patient cohorts [12]. However, bead-based EV capture followed by flow cytometrical analysis has been shown to be a reliable, sensitive and reproducible surface marker detection method and has become readily available [23]. Given this, the present pilot study aimed to employ reproducible and clinically feasible methodology using a standardized bead-based cytometry assay to analyze EVs from periprosthetic joint aspirates. Forthcoming studies may be able to isolate and examine EV surface signatures with little preparation from biological samples [12]. In the future these developments could encourage wider utilization of this tool.

PJI of the knee or hip is a diagnosis with enormous impact on patients and healthcare systems due to the requirement for often times multiple revisions with extended hospitalization [2,24]. Although PJI has a lower incidence than aseptic failure, implant-enabled biofilm development with enhanced bacterial pathogenicity complicates diagnosis and treatment. Currently, there is no reliable “gold standard” for the diagnosis of PJI and instead the decision on treatment is based on a multitude of examinations. Previous reports have specified the potential of EVs in diagnosis as well as treatment of joint diseases and achieved their isolation from synovial fluid of patients with osteoarthritis [14,25,26]. Immune cells, along with the surrounding tissues, may be capable of releasing nanovesicles, which have been demonstrated to possess immunoregulatory functions and been postulated to express compositional variation reactive to bacterial invasion [17,18,19]. These properties could harbor unexploited potential and render them highly interesting for the field of revision endoprosthetics.

To our knowledge the present paper is the first to document the isolation, identification and characterization of EVs from periprosthetic joint aspirates. Electron microscopy provided visual confirmation, while flow cytometry demonstrated the typical expression of CD9, CD63 and CD81—both findings provide evidence of EV existence in our samples. Further, the multiplex result depicts surface epitopes, including CD14, CD24, CD29, CD40, CD41b, CD44, CD49e, CD63, CD81, CD105, HLA-ABC and HLA-DRDPDQ, expressed in both groups while exceeding isotope controls (mlgG1, REA)—indicating that these markers were general vesicle constitutes of periprosthetic joint synovial fluid in our samples.

Nanoparticle tracking analysis was used to determine the overall dimensions and quantity of all isolatable vesicles from artificial joint synovial fluid. This method has been shown to deliver acceptable repeatability and day to day reproducibility for this application [27]. Its results should be interpreted by taking in account the inherent biological sample diversity. These contain a heterogenous group of vesicles of various sizes and are comparable only with caution to preparations from cell culture. Moreover, many measurements displayed higher levels of background noise possibly reflecting the presence of protein aggregates or cellular components other than EVs, that could influence the computation. However, our NTA particle size estimates laid well within EV range and were, in the context of limited comparable literature, similar to Domenis at al. approximation of 172.0 ± 68.4 nm, who isolated EVs from synovial fluid of patients with osteoarthritis via polymer precipitation [15]. Comparing both cohorts, the measured particle sizes were overall greater in AIF cases. Furthermore, our presented quantitative vesicle measurements demonstrated greater amounts within the infected samples, especially in the small EV group (<200 nm). This is consistent with previous studies, that reported increased release of EVs in response to infectious perpetrators and proposed EV count kinetics analogous to the bacterial load in mycobacteria-infected mice [18]. While this study uses ultracentrifugation as a widespread EV isolation methodology, the usage of other more sophisticated techniques such as size exclusion chromatography will be an interesting avenue to pursue and could affect the determined dimensions and quantity of EVs after isolation.

The flow cytometric analysis was based on the presence of archetypal markers CD9, CD63 and CD81, although CD9 was overall comparatively less detected, particularly within the PJI group. Among vesicle subgroups differential expression of these tetraspanins has been described in previous studies, that utilized differential ultracentrifugation and attests to the heterogeneity of the isolated EVs and their subgroups [28]. Particularly CD63 belonged to the highest exhibited markers and comprised together with CD24 the only two epitopes, that where relatively more apparent in PJI samples, which is of high interest due to their immunological functions. Concordantly, a previous report postulated the co-expression of CD63 on CD24-positive B cell microvesicles [29], implying an immunogenic origin of the detected vesicles, that would be expected from the response to bacterial perpetrators. All remaining markers with relevant cytometric fluorescence, showed higher expression rates in the AIF group or were relatively reduced in the PJI group. For example, CD44 and CD105 were likewise significantly less expressed within the PJI group. Both epitopes are indicators for mesenchymal stem cell-derived EVs [30], which have been implicated to possess in vitro immunomodulatory properties on B and T lymphocytes [31,32]. Are these potentially regulatory EVs reduced in PJI cases? Mesenchymal EVs with anti-inflammatory properties may be ordinarily present in periprosthetic joint fluid, but possibly be decreased in a subsequent immunological response to a bacterial infection.

Our results furthermore suggest significantly more HLA-DRDPDQ surface appearance on AIF EVs. This marker has previously been successfully utilized for the isolation of EVs from antigen-presenting cells (APCs) [33]. Although we expected a higher proportion of APC EVs within the PJI samples, our data signifies a contrasting HLA-DRDPDQ EV allocation. This differential distribution of EV-associated HLA-DRDPDQ might affect the process of antigen presentation during particle-induced loosening, which is in line with a differential expression pattern of CD86. One explanation for this discrepancy could be that APC EVs in the infected group may carry peptides for presentation on their MHC II domain [34,35]. In this case the presenting molecular complex undergoes considerable conformational reorientation [36], which leads to redesigned, possibly unfitting molecular surface structures and restricting HLA-DRDPDQ antibody capture within the PJI group. Similarly, CD40 is used for APC activation as an essential co-stimulatory molecule and expected to be more expressed on PJI EVs. However, bacterial presence and associated immunologic molecular adaptations may entail the apparent counter-intuitive lower expression of CD40 on EVs from PJI samples.

Nevertheless, it is crucial to keep in mind that molecular EV compositions do not necessarily resemble distinguishing molecular features of the releasing cell and that the liberated EV’s configuration could be heavily alternated prior to its release [29]. This process can make backtracking of particular molecular markers difficult and decipherment of the various EV alternating conditions would be required for such translational endeavors. While our presented results suggest various differences between EVs from PJI and AIF, their origin, destination and therefore much of their specific purpose remains subject to speculation. Since any cell is capable of ejecting EVs it is too often unclear, which tissues or immune cells are communicating with which other entity. Immunologic roles, such as functions in antigen presentation, have been ascribed to EVs from early on and are an established EV quality [17,37]. Immunologic adaptations, in all probability, are therefore responsible for the above described differences between the two investigated cohorts.

In summary, the present paper provides evidence of EVs in periprosthetic joint aspirates and implies the differential manifestation of multiple EV properties in the immediate vicinity of a prosthetic knee or hip in response to a bacterial stressor. Our results suggest higher EV concentrations, smaller EV sizes and particular EV surface marker signatures in PJI. It is too early to use these pilot data to define their diagnostic value via analysis of their sensitivity and specificity to differentiate PJI from AIF or to understand mechanisms such as possible immune paralysis in chronic PJI. Nevertheless, the discovery of EVs in periprosthetic joint fluid, their characterization and the fact that they present differences between PJI and AIF are exciting avenues, which will change our understanding of the cellular and molecular mechanisms of RTJA. However, investigations of specific EV senders and receivers, subgroups and their cargo are required to further decipher their communication and exploit their potential for clinical application in RTJA.

## Figures and Tables

**Table 1:** 2018 MSIS Criteria for the diagnosis of periprosthetic joint infection (PJI) of the knee or hip by Parvizi et al. [4].

Major Criteria		Decision
*at least one of the following:*		*Infected*
1) Two positive cultures from the same organism		
2) Sinus tract with evidence of communication to the joint or visualization of the prosthesis		
**Preoperative diagnosis—Minor criteria**	**Score**	**Decision**
Elevated serum CRP *or* D-Dimer	2	*≥6 Infected 2–5 Possibly infected 0–1 Not infected*
Elevated ESR (serum)	1	
Elevated synovial WBC count *or* LE	3	
Positive synovial alpha-defensin	3	
Elevated synovial PMN	2	
Elevated synovial CRP	1	
**Inconclusive pre-OP score *or* dry tap**	**Score**	**Decision**
Preoperative score	-	*≥6 Infected 4–5 Inconclusive ≤3 Not infected*
Positive histology	3	
Positive purulence	3	
Single positive culture	2	

**Table 2:** Characteristics and demographics of the patient collective.

Characteristics of the Patient Collective							
Variable	Overall (*n* = 23)		PJI Cohort (*n* = 12)		AIF Cohort (*n* = 11)		*P* Value
Age (yr)	71.4	(15.2)	73.0	(14.4)	68.0	(12.5)	0.228
Gender (female)	13	(56.5%)	4	(33.3%)	9	(81.8%)	*0.036* ^a^
Joint (knee)	17	(73.9%)	8	(66.7%)	9	(81.8%)	0.640
BMI (kg/m^2^)	25.3	(4.7)	26.2	(11.8)	25.3	(6.9)	0.712
Time from most recent surgery (month)	11	(25)	7.5	(22)	14	(21)	0.288
Most recent surgery—revision procedure	15	(65.2%)	9	(75.0%)	6	(54.5%)	0.400
Charlsons Comorbiditiy Index	4	(3)	4.5	(1)	3	(2)	*0.011* ^a^

Data presented as median with interquartile range (IQR) or sum with respective percentage ratio. Kilogram (kg); meter (m); year (yr); aseptic implant failure (AIF); periprosthetic joint infection (PJI); ^a^ statistically significant.
